# Deletion of Sarcolemmal Membrane-Associated Protein Isoform 3 (SLMAP3) in Cardiac Progenitors Delays Embryonic Growth of Myocardium without Affecting Hippo Pathway

**DOI:** 10.3390/ijms25052888

**Published:** 2024-03-01

**Authors:** Taha Rehmani, Ana Paula Dias, Marsel Kamal, Maysoon Salih, Balwant S. Tuana

**Affiliations:** Department of Cellular and Molecular Medicine, Faculty of Medicine, University of Ottawa, 451 Smyth Road, Ottawa, ON K1H 8M5, Canada; trehmani@uottawa.ca (T.R.); adias085@uottawa.ca (A.P.D.); msalih@uottawa.ca (M.S.)

**Keywords:** SLMAP3, STRIPAK complex, Hippo signaling, cardiac morphogenesis

## Abstract

The slmap gene is alternatively spliced to generate many isoforms that are abundant in developing myocardium. The largest protein isoform SLMAP3 is ubiquitously expressed and has been linked to cardiomyopathy, Brugada syndrome and Hippo signaling. To examine any role in cardiogenesis, mice homozygous for floxed slmap allele were crossed with Nkx2.5-cre mice to nullify its expression in cardiac progenitors. Targeted deletion of the slmap gene resulted in the specific knockout (KO) of the SLMAP3 (~91 KDa) isoform without any changes in the expression of the SLMAP2 (~43 kDa) or the SLMAP1 (~35 kDa) isoforms which continued to accumulate to similar levels as seen in Wt embryonic hearts. The loss of SLMAP3 from cardiac progenitors resulted in decreased size of the developing embryonic hearts evident at E9.5 to E16.5 with four small chambers and significantly thinner left ventricles. The proliferative capacity assessed with the phosphorylation of histone 3 or with Ki67 in E12.5 hearts was not significantly altered due to SLMAP3 deficiency. The size of embryonic cardiomyocytes, marked with anti-Troponin C, revealed significantly smaller cells, but their hypertrophic response (AKT1 and MTOR1) was not significantly affected by the specific loss of SLMAP3 protein. Further, no changes in phosphorylation of MST1/2 or YAP were detected in SLMAP3-KO embryonic myocardium, ruling out any impact on Hippo signaling. Rat embryonic cardiomyocytes express the three SLMAP isoforms and their knockdown (KD) with sh-RNA, resulted in decreased proliferation and enhanced senescence but without any impact on Hippo signaling. Collectively, these data show that SLMAP is critical for normal cardiac development with potential for the various isoforms to serve compensatory roles. Our data imply novel mechanisms for SLMAP action in cardiac growth independent of Hippo signaling.

## 1. Introduction

During embryogenesis, the first functional organ is the heart, derived from mesoderm and endoderm due to the expression of cardiogenic transcription factors Nkx2.5, Mef2, GATA, Tbx and Hand that work in tandem to differentiate precardiac cells and to control the expression of cardiac genes to mediate cardiac morphogenesis [1]. Cardiac morphogenesis or cardiogenesis is the stage-by-stage development of the heart during embryogenesis beginning at embryonic day 7.5 (E7.5) in mice, where cardiogenic mesoderm cells coalesce at splanchnic mesoderm to form the cardiac crescent. Cells within the cardiac crescent are divided into a collection of cells known as the first heart field (FHF), which migrate to venous poles to form the linear heart tube (E8.5) [2,3]. A subgroup of cells derived from FHF known as secondary heart field (SHF) migrate caudally to further develop the linear heart tube into the looping heart (E9.5). The FHF cells differentiate and proliferate to form the left atria and ventricle, and the SHF cells migrate anteriorly and posteriorly; the anterior SHF cells give rise to the right ventricle and aorta, while the posterior SHF cells generate the atria and the pulmonary artery in maturing embryos, thus generating the immature four-chambered heart (E10.5), which septate to form mature four-chamber heart by E14.5 [4,5]. During the late stages of cardiac development, immature cardiomyocytes grow and increase cardiac function due to a series of structural, metabolic, and transcriptional modifications in a process referred to as cardiomyocyte maturation [6]. Cardiomyocytes will exit the cell cycle shortly after birth; therefore, cardiac growth is sustained through cardiomyocyte maturation to maintain the growing supply of nutrients and oxygen demands of the growing body [7]. Impacting cardiac development at the morphogenesis or maturation stage can increase the risk of developing congenital heart disease and predisposing individuals to later-stage cardiomyopathies. Therefore, understanding the mechanisms that govern cardiac maturation and/or morphogenesis is of much interest.

**Figure 1:**
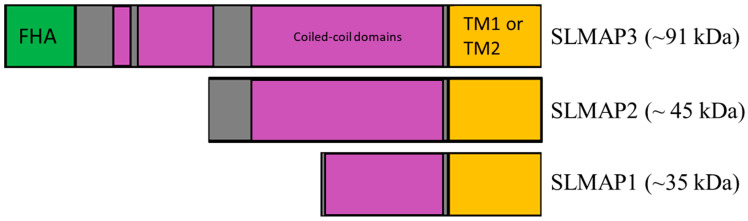
Sarcolemmal membrane-associated protein isoforms and protein domains. SLMAP gene alternatively splices to generate three main protein isoforms, SLMAP1 (~35 kDa), SLMAP2 (~45 kDa), and SLMAP3 (~95 kDa). FHA (green) symbolizes forkhead-associated domain. Coiled-coil domains (purple) identify the coiled-coil leucine zipper domains. TM1 or TM2 (orange) symbolizes the alternatively spliced transmembrane domain 1 or 2.

We have defined the sarcolemmal membrane-associated proteins SLMAP1 (~35 kDa), SLMAP2 (~45 kDa), and SLMAP3 (~80–95 kDa) (Figure 1) that are expressed in myocardium as early as E9 and continue to be accumulated in cardiomyocytes during their maturation and postnatal development [8,9]. The SLMAPs target subcellular organelles including nuclear envelope, endoplasmic/sarcoplasmic reticulum, mitochondria, and the microtubule organizing center (MTOC) [10,11]. The SLMAPs have been shown to associate with cardiac myosin, colocalize with a-actinin and calcium release channels, as well as being found in close apposition to the sarcolemma [9]. Aberrant SLMAP expression has been shown to cause heart failure due to gene mutations present in Brugada patients [12]. Studies indicate that SLMAPs may play roles in regulating the diverse organelles, influence electrophysiology, metabolism, and cardiac function [13,14,15]. However, the role of SLMAPs in the growth and maturation of myocardium remains unknown. The slmap gene is alternatively spliced to generate numerous isoforms that are expressed in a tissue-specific manner, with SLMAP1 and SLMAP2 being abundant in cardiomyocytes [16]. The C-terminus of all SLMAP isoforms comprises an alternatively spliced transmembrane domain, TM1 or TM2, which can target different subcellular membranes, while the extended coiled coil leucine zipper domain and the n-terminal sequences diversify the polypeptide structure for cytoplasmic interactions [10,17]. For example, the SLMAP3 isoform which is ubiquitously expressed carries an n-terminal forkhead-associated (FHA) domain (Figure 1) that can mediate phosphoprotein–protein interactions [11,18] and has been shown to negatively regulate Hippo signaling via the STRIPAK (Striatin-interacting phosphatases and kinases) complex. The Hippo pathway is important for organogenesis through effects on cell proliferation and apoptosis [19]. SLMAP-STRIPAK interactions can inhibit phosphorylation and enhance nuclear translocation of transcriptional co-activators YAP/TAZ and influence TEAD-mediated gene expression [20,21,22]. Hippo signaling has been linked to cardiac development and maturation and can directly impact the size and function of the myocardium [23,24,25,26,27]. SLMAP3 has been reported to regulate the proliferation of cancer cells through Hippo signaling [28]; whether it can impact this pathway to influence growth and development in vivo remains to be examined. Here, we have engineered the specific removal of SLMAP3 from cardiac progenitors to examine its role in cardiogenesis in mice and its impact on the Hippo pathway.

## 2. Results

### 2.1. The Specific Loss of SLMAP3 Isoform in Cardiac Progenitors’ Delays Cardiogenesis

**Figure 2:**
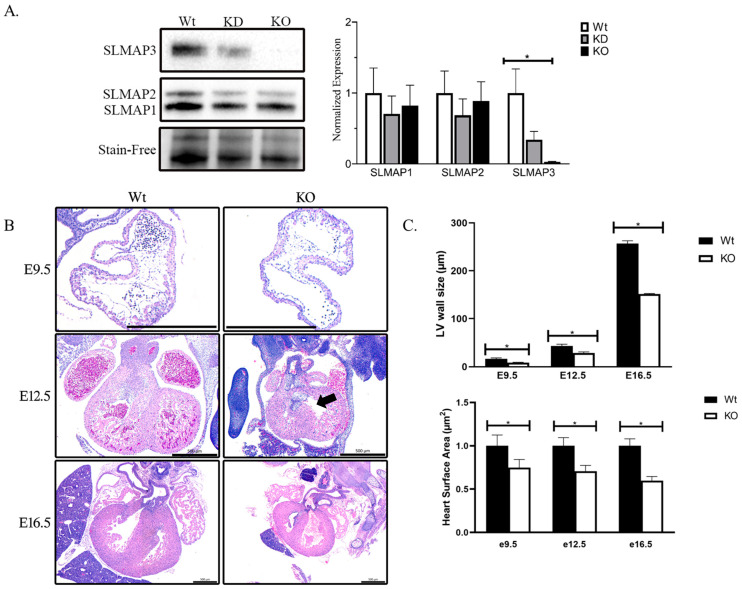
Embryonic heart development in mice with cell-targeted deletion of SLMAP3. (**A**) Western blot displaying SLMAP isoform expression in Wt, KD, and KO in E18.5 mouse heart lysates. Bar graphs represent normalized expression of SLMAP isoforms using Stain-Free™ total protein as a loading control quantified by via densitometry *n* = 6. * ρ < 0.05. (**B**) Representative sections of Wt and KO hearts at E9.5, E12.5, and E16.5 with H&E stain to visualize nucleus (blue) and myocardium (pink) which displayed developing cardiac structures: ventricles, atria, outflow tract, and septa. Black arrow highlights delay during atrioventricular cushion fusion at E12.5 in SLMAP3-KO. Lens magnification = 5×. Scale bar = 0.5 mm. (**C**) Bar graph shows quantification of measurement of left ventricle wall thickness (upper graph) and heart surface area (lower graph) at indicated embryonic ages in Wt and KO hearts. * ρ < 0.05.

We examined the prenatal knockout of the SLMAP gene by crossing floxed SLMAP mice with Nkx2.5-Cre [29] to assess the role of SLMAP during early cardiogenesis. Western blot analysis of E18.5 heart lysates with anti-SLMAP displayed significant loss in SLMAP3 protein levels in (−66.05% ± −15.10%, ρ < 0.05, *n* = 3) heterozygous embryonic hearts (KD) and (−97.36% ± −11.39%, ρ < 0.05, *n* = 3) homozygous (KO) embryonic hearts. No significant changes were noted in the expression levels of SLMAP1 or SLMAP2, indicating the generation of mice with the specific loss of SLMAP3 in fetal myocardium (Figure 2A). We excised embryos at different developmental stages and examined cardiac development (E9.5, E12.5, and E16.5) by cross-section histology with H&E to visualize Wt and SLMAP3-KO myocardium (Figure 2B). Microscopic analysis indicated that prenatal SLMAP3-KO hearts were significantly smaller at each developmental stage E9.5 (−25.16% ± −11.10%, ρ < 0.05, *n* = 3), E12.5 (−29.36% ± −7.86%, ρ < 0.05, *n* = 3) and E16.5 (−40.27%, ± −6.51%, ρ < 0.05, *n* = 3) compared to Wt littermates (Figure 2C). Further, the atria and ventricles appeared to be thinner in SLMAP3-KO, and measurements of the left ventricular walls indicated they were significantly thinner in E9.5 (−48.65% ± −6.61%, ρ < 0.05, *n* = 3), E12.5 (−33.49% ± −5.44 %, ρ < 0.05, *n* = 3), and E16.5 (−41.12%, ± −1.39%, ρ < 0.05, *n* = 3) vs. Wt embryonic hearts (Figure 2C). There is also a noticeable delay in septation in SLMAP3-KO hearts at E12.5 since the atrioventricular cushions have not fused (Figure 2B; black arrow); however, these differences in cardiac chamber formation or septation appeared to be rectified by E16.5 (Figure 2B). Thus, appropriate expression levels of SLMAP3 protein are important to support normal cardiac growth during early cardiogenesis.

### 2.2. STRIPAK and Hippo Signaling Was Unaltered in SLMAP3-Deficient Embryonic Myocardium

**Figure 3:**
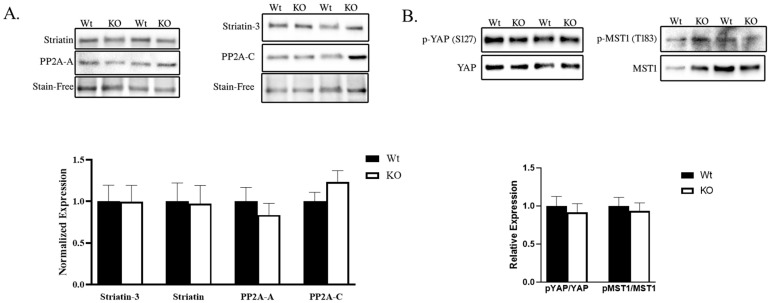
STRIPAK and Hippo signaling in developing myocardium from SLMAP3-depleted cardioprogenetors. (**A**) Western blot showing the expression of STRIPAK components (striatin, striatin-3, PP2A-A, and PP2A-C) in Wt and KO E16.5 hearts. Bar graphs represent normalized expressions of indicated protein to total proteins visualized by Stain-Free™ technology. *n* = 3. (**B**) Western blot showing phospho to total MST1 and YAP in E16.5 hearts. Bar graphs represent relative expression of phospho-YAP (S127) to total YAP and phospho-MST1 (T183) to total MST1 quantified by densitometry. *n* = 3.

Given the dramatic effects of SLMAP3 loss on the growth delay of embryonic heart development and its proposed role as a negative regulator of Hippo signaling via STRIPAK [22,30], we examined any impact on striatin, PP2A-A, PP2A-C, Striatin-3, and the activity of Hippo components in E16.5 Wt and SLMAP3-KO hearts by Western blotting. Striatin (−2.56% ± −22.05%, ρ = 0.93, *n* = 4), striatin-3 (−0.21% ± −19.46%, ρ = 0.89, *n* = 4), PP2A-A (−16.34% ± −16.23%, ρ = 0.54, *n* = 4), and PP2A-C (23.58% ± 11.65%, ρ = 0.20, *n* = 4) were not significantly affected in KO hearts when compared to Wt (Figure 3A). To assess Hippo signaling, we analyzed the phosphorylation of MST1 and YAP; however, the phospho-to-total ratio of MST1 (−6.61% ± −23.56%, ρ = 0.63, *n* = 4) and phospho-to-total ratio of YAP (−8.43% ± −12.25%, ρ = 0.64, *n* = 4) was unaltered in SLMAP3-KO hearts (Figure 3B). Further, qPCR examination for expression of *c-myc* and *CTGF*, which are known downstream gene targets of Wnt and Hippo signaling, showed no significant changes due to SLMAP3 deficiency (ρ = 0.72). Thus, specific deletion of the SLMAP3 isoform in cardiac progenitors delays cardiogenesis but not through any changes in Hippo signaling.

### 2.3. Cardiomyocyte Proliferation Is Not Affected by SLMAP3-KO during Cardiogenesis

**Figure 4:**
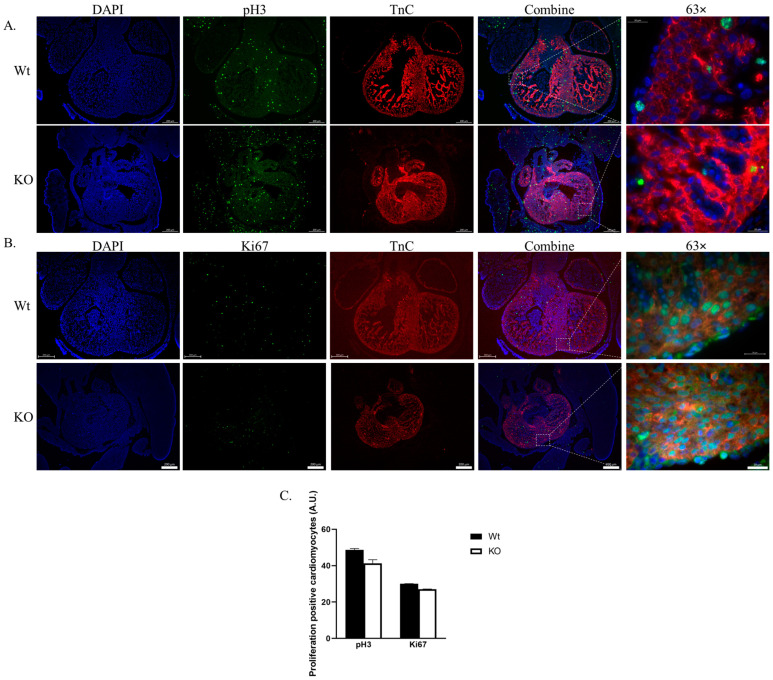
Proliferation of cardiomyocytes in SLMAP3-KO embryonic hearts. Immunofluorescent images of sectioned Wt and KO hearts at E12.5. stained for proliferative marker Histone 3 (**A**) or Antigen Kiel 67 (**B**), nucleus (DAPI; blue), (pH3; green), and anti-troponin C (TnC; red). Lens magnification = 5× or 63×. Representative Scale bar = 0.2 mm at 5× or 0.02 mm at 63×. (**C**) Bar graph representing pH3 or Ki67 proliferation events within TnC^+^-positive cells. *n* = 3.

Cardiac growth is regulated by cardiomyocyte proliferation and hypertrophy through mechanisms involving Hippo and other pathways including FGF, Notch, and Wnt signaling [31]. The evaluation of proliferation with immunofluorescence microscopy for the proliferation markers pH3 and Ki67 in embryos, co-stained with the cardiomyocyte marker Troponin C (TnC) in E12.5 Wt and SLMAP3-KO mouse hearts is shown in Figure 4. The pH3 detects cell division at the G2/M phase (Figure 4A), while Ki67 tags cells at the G1 to G2/M phase of the cell cycle (Figure 4B), providing a cardiomyocyte proliferation index that indicated no significant differences in pH3^+^- or Ki67^+^-positive cardiomyocytes in SLMAP3-KO vs. Wt embryos (Figure 4C). Therefore, the delay in early cardiac development due to the specific loss of SLMAP3 in cardiac progenitors is not due to any changes in their proliferation.

### 2.4. Cardiomyocyte Size Is Reduced in SLMAP3-KO Embryonic Hearts

**Figure 5:**
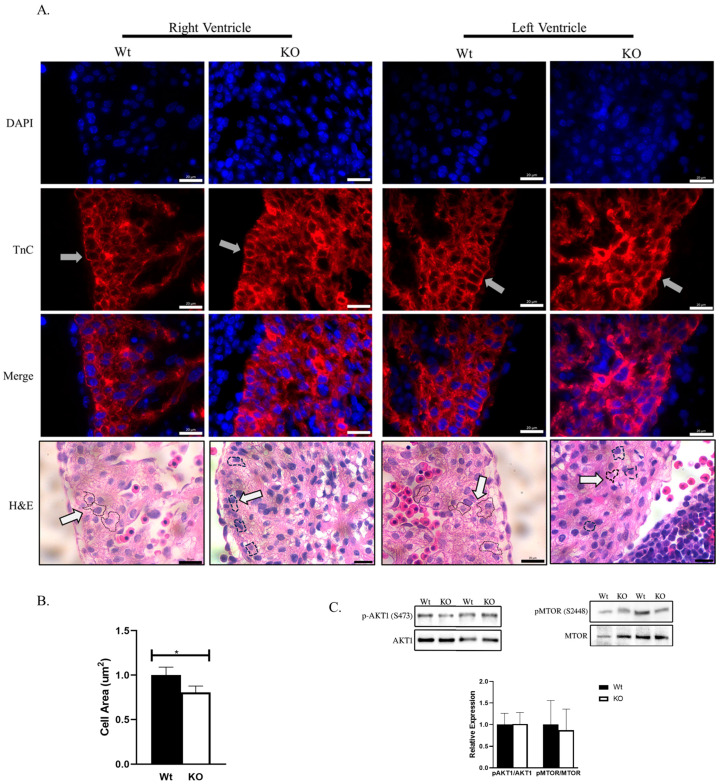
Cell size and hypertrophy in Nkx2.5/SLMAP3-KO mice. (**A**) Images of sectioned Wt and KO hearts at E12.5 showing cradiomyocytes from right and left ventricles stained for the nucleus (DAPI; blue) and cardiac anti-troponin C (TnC; red) or H&E (pink; myocardium). Black dash lined indicates the perimeter and shape of a cardiomyocyte marked by white arrow. Lens magnification = 63×. Scale bar = 0.02 mm. (**B**) Bar graph shows quantification of left ventri cular cardiomyocyte area in Wt and KO E12.5 embryonic hearts. * ρ < 0.05, *n* = 3. (**C**) Western blot showing expression of phospho-to-total ratios of AKT1 and mTOR in E16.5 Wt and KO hearts. Bar graphs represent quantification of relative expression of phospho-mTOR (S2448) to total mTOR and phospho-AKT1 (S473) to total AKT1 with densitometry of the Western blots. *n* = 3.

Since changes in proliferation could not account for the smaller embryonic hearts, we investigated if the size of cardiomyocytes was affected by the loss of SLMAP3 by assessing right ventricle and left ventricle cardiomyocytes in E12.5 heart sections, and the magnified images obtained with H&E and TnC staining is shown (Figure 5A). We identified the perimeter of cardiomyocytes with H&E (black dashed line, white arrow) and cardiomyocytes with TnC (grey arrow) to calculate the area that represents the cardiomyocyte size. Our analysis on left ventricular cardiomyocytes appeared to be significantly smaller in KO (−19.43% ± −7.55%, ρ < 0.05, *n* = 3) when compared to Wt (Figure 5B). The cardiomyocytes also appeared to be disorganized in SLMAP-KO E12.5 hearts where the nuclei of neighboring cardiomyocytes cluster together with changes in cardiomyocyte shape and orientation (Figure 5A, grey arrows). Cardiomyocyte hypertrophy is linked to the upregulation of AKT1 and MTOR1 [32], which affect cell survival and growth. The activity of AKT1 and MTOR1 assessed in Western blots indicated that the phospho-to-total ratios of AKT1 (1.45% ± 26.50%, ρ = 0.97, *n* = 3) and mTOR (−12.87% ± −51.12%, ρ = 0.64, *n* = 3) were not significantly altered in E16.5 SLMAP3-KO hearts compared to Wt (Figure 5C). Thus, the specific loss of SLMAP3 during cardiogenesis leads to significant changes in the size and structure of cardiomyocytes but without any impact on the hypertrophic program.

### 2.5. SLMAP3-KO Hearts Grow to Normal Size and Function in Postnatal Development

**Figure 6:**
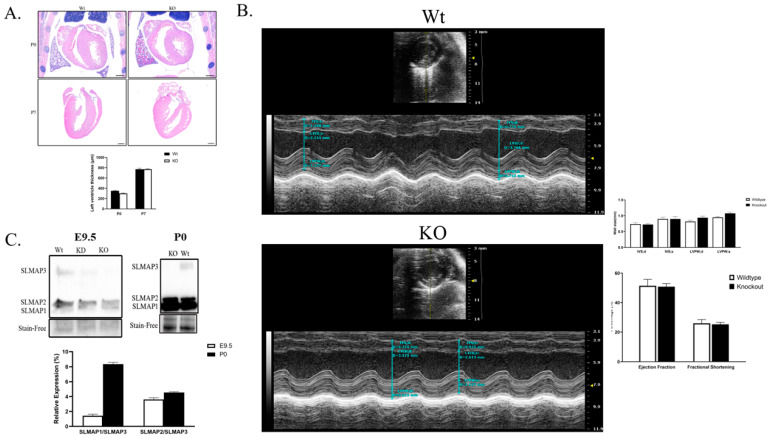
Analysis of postnatal hearts from Nkx2.5-Cre/SLMAP3-null mice. (**A**) Representative histological staining of Wt and KO Nkx2.5-Cre/SLMAP3-null myocardium at P0 and P7 with H&E. Scale bar = 0.5 mm. (**B**) Representative short axis echocardiography m-mode images of 10-week-old Wt and KO. Bar graphs show quantification of ejection fraction, fractional shortening, and left ventricle wall thickness. LV, left ventricle; IVS, LVPW, intraventricular septum, LV posterior wall; d, s, diastole, systole. *n* = 6. (**C**) Western blot displaying expression of SLMAP isoforms (SLMAP1, SLMAP2 and SLMAP3) in Wt and KO hearts at E9.5 and P0. Bar graph is the quantification of relative expression of SLMAP1 or SLMAP2 compared to SLMAP3 in E9.5 and P0 Wt hearts. *n* = 3.

Despite the significant delay in the growth of the embryonic myocardium, the cardiac-specific SLMAP3-KO neonatal pups were born normally without any obvious cardiac phenotypes at birth and survived into adulthood similar to Wt. We sectioned P0 and P7 SLMAP3-KO and Wt hearts and stained them with H&E to visualize cardiac structure. At postnatal day 0 (P0); the left ventricle size of KO hearts (−14.19% ± −7.55%, ρ = 0.10, *n* = 3) was almost similar to Wt hearts (Figure 6A). At P7, the H&E stain revealed no differences in the left ventricle wall size in KO hearts (−0.08% ± −0.7 8%, ρ = 0.97, *n* = 3) (Figure 6A). Further, examination of adult SLMAP3-KO with ECHO at 10 weeks of age indicated no significant differences in cardiac structure or function (Figure 6B) with an equivalent survival age to Wt.

We quantified the expression of SLMAP isoforms in SLMAP3-KO hearts, and Western blot analysis indicated no changes in SLMAP1 and SLMAP2 protein levels in KO vs. Wt at E9.5 or P0, and SLMAP3 protein was not detected in the KO at either stage. However, the relative expression of SLMAP1/SLMAP3 and SLMAP2/SLMAP3 in the wild-type myocardium was 6.07× and 1.42× greater in P0 hearts when compared to E9.5 (Figure 6C). The data indicate that as the myocardium develops, there is a substantial increase in the expression of the smaller SLMAP isoforms compared to SLMAP3, and the loss of SLMAP3 did not influence their expression levels in embryonic or postnatal myocardium.

### 2.6. Depletion of SLMAP Isoforms in Rat Embryonic Cardiomyocytes (H9C2 Cells) Impacts Their Growth

**Figure 7:**
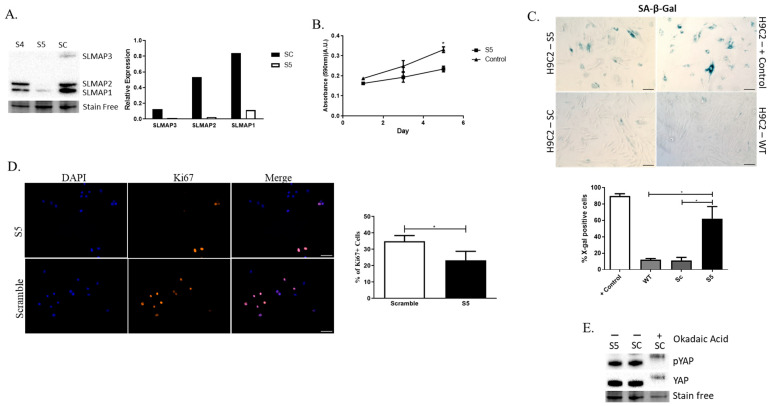
Loss of SLMAP expression in embryonic cardiomyocytes impacts growth and senescence. (**A**) Western blot displaying expression of SLMAP1, SLMAP2, and SLMAP3 in sh-RNA transduced H9C2 cells and bar graph showing quantification of expression of SLMAPs in Sc, S4, and S5 cells normalized to total proteins visualized by Stain-Free^TM^. (**B**) Line graph displaying crystal violet absorbance measurements on days 1, 3, and 5 after seeding transduced H9C2 to compare growth rates. * ρ < 0.05. (**C**) Senescence analysis on Wt positive (+) control, Wt, and transduced (Sc, S5) H9C2 cells with SA-β-Gal assay. Bar graph represents percentage (%) of X-gal-positive cells. * ρ < 0.05. Scale bar = 100 µm. (**D**) Immunofluorescence staining with DAPI (Blue; nucleus) and Ki67 (orange) in transduced H9C2 cells. Bar graph represents mean percentage of Ki67^+^ cells. * ρ < 0.05. Scale bar = 100 µm. (**E**) Western blot evaluating phospho to total YAP in transduced H9C2 and Sc-H9C2 cells treated with okadaic acid as a positive control.

To examine whether all three isoforms expressed in the heart are important for the growth of the embryonic myocardium, we investigated the expression of SLMAPs in the rat embryonic cardiomyocyte cell line H9C2. Western blot analysis indicated that H9C2 cells express the three SLMAP isoforms-SLMAP-1, 2, and 3 (Figure 7A), identical in molecular size to that seen in mouse embryonic hearts (Figure 6C). H9C2 cells transduced with shRNA-S4 (S4) or shRNA-S5 (S5) displayed a decrease in SLMAP expression when compared to the control scrambled shRNA-Sc (Sc), although only S5 drastically reduced the expression of all three SLMAP isoforms and was employed in all subsequent studies (Figure 7A). Notably, the depletion of all SLMAP isoforms in H9C2 cells resulted in a decrease in growth rate (Figure 7B). Further, the depletion of SLMAP isoforms lead to an increase in X-gal-positive cells in the Senescence-Associated-β-Galactosidase (SA-β-Gal) Assay (Figure 7C), and a decrease in proliferation, as indicated by the reduced number of Ki67-positive cells (Figure 7D). Western blots of transduced H9C2 lysates indicated that the depletion of SLMAPs had no impact on the phosphorylation of YAP in these embryonic cardiomyocytes (Figure 7E). Thus, SLMAP isoforms appear to play an important role in the growth of embryonic cardiomyocytes, independently of Hippo signaling.

## 3. Discussion

Here, we report that the specific loss of SLMAP3 isoform in cardiac progenitors by gene targeting with Nkx2.5-cre mice leads to a growth delay in the formation of the heart during early cardiogenesis. The developmental delay in cardiac formation was not due to changes in the proliferation or hypertrophic response in embryonic cardiomyocytes, although a difference in cell size was evident at E12.5 due to SLMAP3 deletion. Intriguingly, the early delay in cardiogenesis was completely reversed at birth and the postnatal development and function of the heart was unaffected in these mice in the absence of any SLMAP3. While the expression of SLMAP3 protein was completely nullified in cardiac progenitors, due to Nkx2.5-cre, the expression of the more abundant SLMAP1 and SLMAP2 remained unchanged and continued to accumulate normally during embryonic and postnatal development to levels that were similar to those in wildtype mouse hearts. SLMAP2 and SLMAP1 expression levels remained unaffected, most likely due to the utilization of alternative promoters in cardiomyocytes (Salih and Tuana, unpublished data). Whether SLMAP1 and SLMAP2 can compensate for a loss of SLMAP3 function needs to be considered because of the very close structural similarities between these isoforms, which are derived by alternative splicing mechanisms from a single gene (Figure 1). In this regard, SLMAP3 is clearly distinguishable from the other SLMAPs due to its extended N-terminal sequences which encompass an FHA domain that was shown to bind MST1/2 kinases and regulate Hippo to impact proliferation of 293 T cells [30]. The in vivo data here did not support a role for SLMAP3 in the proliferation of embryonic cardiomyocytes or the regulation of MST or YAP phosphorylation, since SLMAP3 deletion had no impact on these parameters during cardiogenesis. The loss of SLMAP3 did not affect Hippo signaling in the postnatal heart either, and the myocardium appeared normal in terms of its structure and function in adulthood. These data are consistent with our studies on the targeted deletion of the SLMAP3 isoform in postnatal myocardium [33]. Our previous data have shown that SLMAP is expressed early during cardiogenesis, and thus could influence the growth and maturation of cardiomyocytes. Perhaps the deletion of the other SLMAP isoforms is necessary to observe the critical nature of SLMAP in developing myocardium. Cardiac development begins at E7.5 in mice with the formation of the cardiac crescent, when the cardiac homeobox transcription factor Nkx2.5 is highly expressed within cardiac progenitor cells [34]. Nkx2.5-Cre mice are often employed to study genes during early cardiogenesis [29,35], and the deletion of SLMAP3 expression at this stage within FHF and SHF did not apparently impact cardiomyocyte differentiation/migration; however, there was a growth delay in septation with underdeveloped cardiac chambers in these mice hearts at E12.5 but without any obvious structural abnormalities. In order to interrogate the potential role of the other SLMAP isoforms, we employed rat embryonic cardiomyocytes (H9C2 cells) that were found to express SLMAP1, SLMAP2, and SLMAP3 with similar molecular characteristics to those in mouse embryonic heart tissue. Our constructed sh-RNAs-targeting SLMAPs resulted in a significant decrease in the expression of the three SLMAP isoforms accompanied by a profound deficit in growth and proliferation as well as enhanced cellular senescence in these embryonic cardiomyocytes. It is notable that depletion of the three SLMAPs in H9C2 cells had no impact on the main components of Hippo signaling. Thus, the mechanisms of SLMAP action that impact the embryonic growth of cardiomyocytes are independent of any regulation through Hippo signaling. The data support the contention that SLMAP isoforms are collectively critical for embryonic cardiomyocyte growth and that they can potentially compensate for each other since the specific embryonic KO of the SLMAP3 isoform in early heart development was non-lethal. It is conceivable that the more abundant SLMAP1 and SLMAP2 proteins may substitute for the loss of SLMAP3 during cardiogenesis. Given that the knockdown of all three SLMAP isoforms in H9C2 cardiomyocytes lead to defective growth and cellular senescence, it would be meaningful to delete SLMAP1 and SLMAP2 in cardiac progenitors in vivo to gauge the contribution of SLMAP to heart development, although the H9C2 data would suggest that it would be lethal. The size of cardiomyocytes was noticeably smaller in embryonic hearts due to Nkx2.5-Cre-mediated SLMAP3 loss, but there was no change in hypertrophic signaling (AKT/mTOR), and nor was there any change in cardiomyocyte numbers. We aim to employ additional stereological analysis by isolating embryonic cardiomyocytes and further staining to decipher the mechanisms that may impact their size and shape at the cellular level.

It is notable that unique structural elements in SLMAP3 (i.e., FHA domain) target the MTOC [11], which has been shown to be critical for mitosis and differentiation [36]. The microtubules and the cytoskeleton serve important roles in cell shape and function, and their contributions to cardiomyocyte growth and size/shape in vivo remain elusive. The data here further point to a unique structural mechanism for SLMAP actions through their interactions with subcellular organelles, microtubules, and the cytoskeleton to guide cardiac development.

## 4. Materials and Methods

### 4.1. Generating and Genotyping the SLMAP Knockout Model

The Nkx2.5-Cre mice [29] were generously donated by Dr. Kyoung-Han Kim and backcrossed with wildtype B6C3F1 mice to generate the B6C3F1 Nkx2.5-cre mice. The generation of our flox-SLMAP, breeding strategies, and PCR genotyping were performed as described previously [33]. Briefly, B6C3F1 flox-SLMAP mice were crossed with B6C3F1 Nkx2.5-cre mice to generate heterozygous floxed-SLMAP;Nkx2.5-cre or knockdown (KD) mice. Crossing KD mice with each other generates homozygous floxed-SLMAP;Nkx2.5-cre or knockout (KO) mice. Mice harboring heterozygous or homozygous Nkx2.5-cre were followed for one year and did not exhibit any abnormalities. Thus, chronic expression of cre did not result in any phenotypes.

For embryo studies, pregnant mothers that were plug-checked (day of plug was E0.5) and carrying our experimental embryos at the desired stage were anesthetized with a standard ketamine–xylazine–acepromazine (KXA) drug cocktail during embryo isolation and euthanized after they were harvested. The embryos were dissected in ice cold 1x PBS and whole embryos or prenatal hearts were fixed in 10% neutral buffered formalin for 48–96 h or snap-frozen in liquid nitrogen for proteomic analysis. Caudal tissues or tails were used for genotyping.

### 4.2. Protein Isolation and Western Blotting

Hearts of embryonic (E9.5–E18.5) or P0 mice were collected after explanting from mothers and immediately frozen at −80 °C. Each heart was later washed with ice-cold 1× phosphate buffered saline (PBS) and homogenized using a Fisher handheld Maximizer homogenizer (Thermo Fisher Scientific, Waltham, MA, USA) in ice-cold lysis buffer (1 mM ethylene glycol tetraacetic acid (EGTA), 1 mM ethylenediaminetetraacetic (EDTA), 20 mM Tris base, 1% Triton, 150 mM sodium chloride, 1× complete mini EDTA-free protease inhibitor cocktail (Roche, Basel, Switzerland), and 1× PhosSTOP (Roche). The suspension was centrifuged for 15 min at 12,000× *g* to separate the proteins from the cell debris. The supernatant-containing protein was stored at −80 °C. Protein concentration was calculated using the BSA method.

For Western blotting experiments, 10 µg of protein was loaded in each well of a 4–15% gradient SDS-PAGE gel. The gels were transferred overnight on a polyvinylidene fluoride (PVDF) membrane (Bio-Rad) in a buffer containing 25 mM Tris, 190 mM Glycine, and 20% methanol. All membranes were blocked at room temperature for 1 h in Tris-buffered saline (TBST) containing 1 M Tris, 290 mM NaCl, 0.1% Tween 20, pH 7.4, and 5% bovine serum albumin. Primary antibodies (listed in Table 1) were incubated overnight at 4 °C with 5% bovine serum albumin (BSA). Membranes were washed 3 times for 5 min each in TBST prior to adding the appropriate horseradish peroxidase-labeled secondary antibody (Jackson ImmunoResearch, West Grove, PA, USA) in a 1:10,000 dilution in TBST with 5% nonfat dry milk. Membranes were shaken slowly at room temperature for 1 h while incubating with secondary antibody, followed by 3 washes for 5 min each with TBST. Membranes were treated with a BioRad Western blotting kit (Bio-Rad, Hercules, CA, USA) and developed using ChemiDoc machines (Bio-Rad, Hercules, CA, USA). Bands were quantified by densitometry using Image Lab software v.6.0.0 (Bio-Rad). Membranes were stripped (25 mM glycine, 10% SDS, and pH 2.2 in dH_2_O) and reprobed with different antibodies. When using stain-free technology, stain-free gels (Bio-Rad) and low-fluorescence PVDF membranes (Bio-Rad) were used.

### 4.3. Echocardiography

All echocardiographic analysis was performed using the VEVO 2100 system (FUJIFILM VisualSonics), identically to our previous publication [33].

### 4.4. Histological and Immunofluorescent Analysis

Embryonic and postnatal mouse hearts were fixed using 10% Neutral Buffered Formalin (Thermo Fisher Scientific). After fixing for 48 h, hearts were sectioned 4 µm longitudinally per section. Paraffin-embedded slides were rehydrated through well-established protocols for rehydration with citrate buffer for antigen retrieval [37]. For histological analysis, sectioned hearts were stained with hematoxylin and eosin (H&E) stain to visualize the myocardium and nuclei. For immunofluorescence, rehydrated slides were subjected to 1 h of 5% BSA in PBS-T (1× PBS, 0.01% Triton X-100) of blocking. After rinsing with PBS, we diluted antibodies according to Table 1 in blocking solution and incubated them overnight at 4 °C. Samples were then washed 3x for 5 min with PBS, and then secondary Alexa-555 (Thermo Fisher Scientific, USA) or Alexa 647 (Thermo Fisher Scientific, USA) were incubated for 1 h at room temperature. We used VectaShield Plus DAPI (MJS Biolynx, Brockville, ON, Canada) mounting medium with 1.5 mm coverslips (Fisher Scientific, USA) sealed with nail polish and imaged when completely dried. Images were captured on Zeiss AxioImager M2 and analyzed using ImageJ v1.8.

Cardiomyocyte size was measured by staining sectioned E12.5 hearts with H&E or immunofluorescence for troponin C (TnC). We marked the perimeter of cardiomyocytes based on the staining and used ImageJ v1.8 to calculate the area of cardiomyocytes which would reflect their size.

### 4.5. Creation of Short-Hairpin RNA (sh-RNA) Lentivirus for Depletion of SLMAP3

To stably deplete SLMAP3 expression in H9C2 cells, we designed two shRNAs targeting mouse SLMAP sequences from exon 22 (Ensembl reference transcript ENSMUST00000139075.8), which were conserved in rat SLMAP gene. The targeting sequences are 5′-GCAATCAATCACAGATGAGCTCAAA and 5′-GCTGCTGCGAGAGAAAGGAAATAAT, which were called S4 and S5, respectively. The control scramble target sequence is 5′-AGGATAAGCGTCAACGAATAGGTGA, referred to as SC, and was generated by inputting the S5 plasmid sequence into GenScript Sequence Scramble tool [38]. The shRNAs were designed in a lentivirus vector with the VectorBuilder design tool [39]. VectorBuilder cloned and delivered the plasmids in *Escherichia coli* (VB UltraStable). Plasmids were isolated via PureYield Plasmid Midprep System protocol (Promega Cooperation, Madison, WI, USA, Technical Manual #TM253) and transfected in Lenti-X 293 T cells with the packing and envelope plasmids psPAX2 [40] and pMD2.G [41] via polyethyleneimine (PEI), following an established protocol [42]. The supernatants of Lenti-X 293 T cells containing lentiviruses were harvested 48 and 72 h after transfection and filtered with 0.45 µm sterile syringe filter. The viral particles were concentrated via Lenti-X-Concentrator (Clontech, Mountain View, CA, USA, catalog number PT4421-2), and the titer was quantified with the PCR Lentivirus Titer Kit (abm, New York, NY, USA, catalog number LV900).

### 4.6. Maintenance and Transduction of H9C2 Rat Cardiomyocyte Cell Line

H9C2 cell line was originally acquired from ATCC and gifted by the Megeney Laboratory at University of Ottawa. H9C2 cells were kept at 37 °C with 5% carbon dioxide in high humidity. H9C2 media consisted of high-glucose (4.5 g/L) Dulbecco modified essential media (DMEM) (Multicell, Woonsocket, RI, USA, Catalogue #319-005-CL) containing 1.00% pen-strep, 1.00% non-essential amino acids (Gibco, Carlsbad, CA, USA, Reference #11140-050). The H9C2 cell line was tested in the lab for mycoplasma contamination and tested negative in all instances. The lentiviral reverse transduction was performed following Addgene protocol [43]. H9C2 cells were plated at 25,000 cells per cm^2^ of growth area and transduced with S4, S5, and Sc at multiplicity of infection (MOI) of 150. Selection for successfully transduced cells was carried out by incorporating puromycin into cell media (3 μg/mL) for 3 days. For induction of YAP phosphorylation to be used as a positive control, H9C2 cells were treated with 1.5 μM okadaic acid for 1 h. Protein lysate was isolated soon after that.

### 4.7. Senescence Analysis on H9C2 

Cellular senescence was analyzed via Senescence Beta-Galactosidase Staining Kit according to the protocol outlined by the manufacturer (Cell Signaling Technology, Danvers, CO, USA, Cat. #9860). All the cells were plated in triplicate, and cells were quantified in 3 fields of each. Total and positive X-gal cells were counted blindly to avoid any bias. For positive control, we induced cellular senescence in H9C2 WT cells by 600 μm H_2_O_2_ treatment for 2 h in two consecutive days.

### 4.8. H9C2 Growth Curve Analysis

Cells were plated at 9000 cells per cm2 and left overnight to attach. Cells were fixed with 4% paraformaldehyde in 1X PBS, pH 7.4 (warmed to 37 °C) for 20 min followed by distilled water wash. Samples were incubated with 0.1% crystal violet for 20 min at room temperature followed by 4 distilled water washes. Samples were left to air dry at room temperature for a minimum of 2 h followed by 10% acetic acid incubation for 20 min at room temperature, while shaking, to dissolve the crystal violet. Quantification was performed by measuring absorbance of samples at 590 nm relative to each other using Synergy Hybrid Multi-Mode Microplate Reader (BioTek Instruments, Highland Park, VT, USA). Procedure was performed 3 times corresponding to the day of the experiment (days 1, 3, and 5) for each sample.

### 4.9. Statistical Analyses

The statistical analysis was performed using GraphPad Prism software version 8 for Windows (GraphPad Software, San Diego, CA, USA). All comparisons between wildtype and knockout groups were analyzed using non-paired two-tailed Student’s *t*-test. If three groups were analyzed, we used one-way ANOVA with post hoc Tukey test to determine significance. All values and points on graphs represent mean values obtained from multiple experiments. All error bars presented in graphs are represented using the standard error of the mean. All sample size values (*n*) represent replicates.

## Figures and Tables

**Table 1:** List of antibodies used in this study. All antibodies used in this study are listed with the corresponding distributor, catalog number, and dilution used for Western blot (WB) or immunofluorescence (IF). Novus Biological (Centennial, CO, USA), BD Transduction Laboratories (Franklin Lakes, NJ, USA), Bethyl Laboratories (Montgomery, TX, USA), Santa Cruz (Dallas, TX, USA), Cell Signaling Technology (Danvers, MA, USA), Abcam (Cambridge, UK).

Antibody	Manufacturer	Dilution
SLMAP	Novus Biologicals (NBP1-81397)	1:1000 (WB)
Striatin-1	BD Transduction Laboratories (610838)	1:1000 (WB)
Striatin-3	Novus Biological (NBP-74572)	1:1000 (WB)
STRIP1	Bethyl Laboratories (A304-644A)	1:1000 (WB)
PP2A-C α/β subunit	Santa Cruz Biotechnology (sc-80665)	1:200 (WB)
PP2A-A α subunit	Millipore Sigma (07-250)	1:600 (WB)
Phospho-YAP (S127)	Cell Signaling Technology (13008S)	1:1000 (WB)
YAP	Cell Signaling Technology (14074S)	1:1000 (WB)
pH3	Abcam (ab5176)	1:200 (IF)
Ki67	Abcam (ab15580)	1:200 (IF)
Troponin C (TnC)	Santa Cruz (sc-52265)	1:200 (IF)
pAKT1	Cell Signaling Technology (9018S)	1:1000 (WB)
AKT1	Cell Signaling Technology (9272)	1:1000 (WB)
pMTOR1	Cell Signaling Technology (5536)	1:1000 (WB)
MTOR1	Cell Signaling Technology (2972)	1:1000 (WB)

## Data Availability

Data are contained within the article.
